# Elucidating Sorghum Biomass, Nitrogen and Chlorophyll Contents With Spectral and Morphological Traits Derived From Unmanned Aircraft System

**DOI:** 10.3389/fpls.2018.01406

**Published:** 2018-10-02

**Authors:** Jiating Li, Yeyin Shi, Arun-Narenthiran Veeranampalayam-Sivakumar, Daniel P. Schachtman

**Affiliations:** ^1^Department of Biological Systems Engineering, University of Nebraska-Lincoln, Lincoln, NE, United States; ^2^Department of Agronomy and Horticulture, University of Nebraska-Lincoln, Lincoln, NE, United States

**Keywords:** UAV, phenotyping, multispectral, nitrogen stress, biomass, chlorophyll, plant height, canopy cover

## Abstract

Unmanned aircraft systems (UAS) provide an efficient way to phenotype crop morphology with spectral traits such as plant height, canopy cover and various vegetation indices (VIs) providing information to elucidate genotypic responses to the environment. In this study, we investigated the potential use of UAS-derived traits to elucidate biomass, nitrogen and chlorophyll content in sorghum under nitrogen stress treatments. A nitrogen stress trial located in Nebraska, USA, contained 24 different sorghum lines, 2 nitrogen treatments and 8 replications, for a total of 384 plots. Morphological and spectral traits including plant height, canopy cover and various VIs were derived from UAS flights with a true-color RGB camera and a 5-band multispectral camera at early, mid and late growth stages across the sorghum growing season in 2017. Simple and multiple regression models were investigated for sorghum biomass, nitrogen and chlorophyll content estimations using the derived morphological and spectral traits along with manual ground truthed measurements. Results showed that, the UAS-derived plant height was strongly correlated with manually measured plant height (*r* = 0.85); and the UAS-derived biomass using plant height, canopy cover and VIs had strong exponential correlations with the sampled biomass of fresh stalks and leaves (maximum *r* = 0.85) and the biomass of dry stalks and leaves (maximum *r* = 0.88). The UAS-derived VIs were moderately correlated with the laboratory measured leaf nitrogen content (*r* = 0.52) and the measured leaf chlorophyll content (*r* = 0.69) in each plot. The methods developed in this study will facilitate genetic improvement and agronomic studies that require assessment of stress responses in large-scale field trials.

## Introduction

Following rice, wheat, corn, and barley, sorghum is the fifth most important cereal crop worldwide (Ramatoulaye et al., 2016). It is widely used in human consumption, animal feed, and biofuel production (Stanton et al., 2017). As reported, in 2016, the sorghum production in the U.S. was about 12.2 million tons which is approximately 20% of the world sorghum production (63.93 million tons) (FAOSTAT, 2017). Serving as the biomass crop for biofuel production, sorghum has the advantages of an annual growth cycle, high caloric value, and low management cost (Fernandes et al., 2018). An efficient and timely method for the prediction of sorghum biomass will help to speed the development of higher biomass varieties. The benefits of sorghum as a biomass crop could be further enhanced if genotypes with high tolerance to stresses such as reduced nitrogen or water deficit can be more easily identified, which will be facilitated by integrating sorghum genotyping and phenotyping technologies.

In the past decade, gene sequencing technology has advanced, allowing the crop genomic information to be collected much easier and more cheaply (Furbank and Tester, 2011). However, genomic selection is still hampered by the speed and ease of obtaining large amounts of phenotypic information. Traditionally, in-field phenotyping has been conducted manually, which consumes a great deal of labor and time. High-throughput phenotyping technology developed in recent years opens opportunities to automate and speed up breeding pipelines. Depending on the traits of interest and growth stages, high-throughput phenotyping can be conducted either in the lab or in the field. For the field-based phenotyping, the ground-based systems and the aerial-based systems usually work as complementary platforms to achieve the final goal of rapid and accurate trait collection. Ground-based systems such as the gantry systems (Virlet et al., 2017), cable-suspended systems (Kirchgessner et al., 2017) and mobile cart or robotic systems (Svensgaard et al., 2014) conduct proximal sensing over or under the plant canopy with little limitation on sensor weight or size. The aerial high-throughput phenotyping usually implemented with unmanned aircraft systems (UAS) operated at low altitudes which have limited sensor payloads or weight and can only detect traits remotely over the canopy. However, they are capable of covering a larger area in a shorter period of time which minimizes the measurement error caused by changes in environmental factors, and are independent of the soil condition which may hamper movement of ground based systems. Typical types of UAS are fixed-wing, rotary-wing, and hybrid systems. A rotary-wing platform was selected in this study to conduct slow speed, low altitude and more stable phenotypic data collection for sorghum.

UAS technology has been widely used to study various traits in different crops including sorghum. Morphological traits are often measured from natural color images, i.e., RGB images, or estimated from spectral images. Sorghum and corn plant height is a trait that has been investigated in several studies using the structure from motion technique and RGB images (Shi et al., 2016; Watanabe et al., 2017; Hu et al., 2018; Malambo et al., 2018; Pugh et al., 2018). Sorghum ground cover (Duan et al., 2017; Potgieter et al., 2017; Shafian et al., 2018) and leaf area index (Potgieter et al., 2017; Shafian et al., 2018) were directly calculated from the RGB images or estimated using spectral information from multispectral camera. Visible morphological traits are easier to measure than physiological traits, such as chlorophyll content, nitrogen concentration, and water content. The physiological traits are often hard to be assessed by the human eye but can be detected in the infrared spectra and the variations in those important traits become more obvious if they are depicted using vegetation indices (VIs). For example, normalized difference red edge (NDRE) was used to differentiate stay-green and senescent lines in sorghum breeding (Potgieter et al., 2017). Sorghum grain yield was well correlated with the normalized difference vegetation index (NDVI) derived from a modified three-band camera (green, red, and NIR) (Stanton et al., 2017) and a multispectral camera (Shafian et al., 2018). Sorghum panicle volume was estimated from RGB orthomosaic, DSM and point cloud (Chang et al., 2017a).

Biomass and nitrogen status of sorghum is particularly important for the development of new higher yielding nitrogen use efficiency energy sorghum varieties for lignocellulose production. While grain sorghum is easy to harvest, energy sorghum is not because of its very large size. The main interest of growing energy sorghum is in the biomass which may be used to produce cellulosic ethanol. The crop is over 3.5 to 4.5 m high and specialized equipment which is not usually readily available is required for harvest. Therefore, the use of UAS to estimate biomass and nitrogen status of sorghum provides a highly efficiency way for breeders to improve the crop. Most of the UAS related studies on sorghum were focused on plant height, ground cover, leaf area index and grain yield estimation so far. The only study we found for sorghum biomass estimation was using a UAS based hyperspectral and RGB system and machine learning modeling (Zhang et al., 2017). The results were promising which inspired us to move forward to investigate alternative low-cost method based on multispectral and RGB cameras for sorghum biomass, nitrogen, and chlorophyll content estimation. As for sorghum nitrogen and chlorophyll estimation, most of the previous studies focused on qualitative differentiation between treatments such as high and low nitrogen treatment or stay-green and senescent lines; while no study was found that investigated the quantitative relationship between sorghum nitrogen or chlorophyll content and UAS-derived traits.

The objective of this study was to investigate the potential of using UAS-derived multispectral and morphological traits for sorghum biomass, nitrogen and chlorophyll content estimates. Three specific objectives were:

1. Obtain sorghum spectral and morphological traits from UAS based remote sensing, including various vegetation indices, plant height and canopy cover;
2. Establish predictive models for sorghum biomass, nitrogen and chlorophyll contents using the obtained morphological and spectral traits; and
3. Evaluate how predictions compared with ground truth measurements.

## Materials and methods

**Figure 1:**
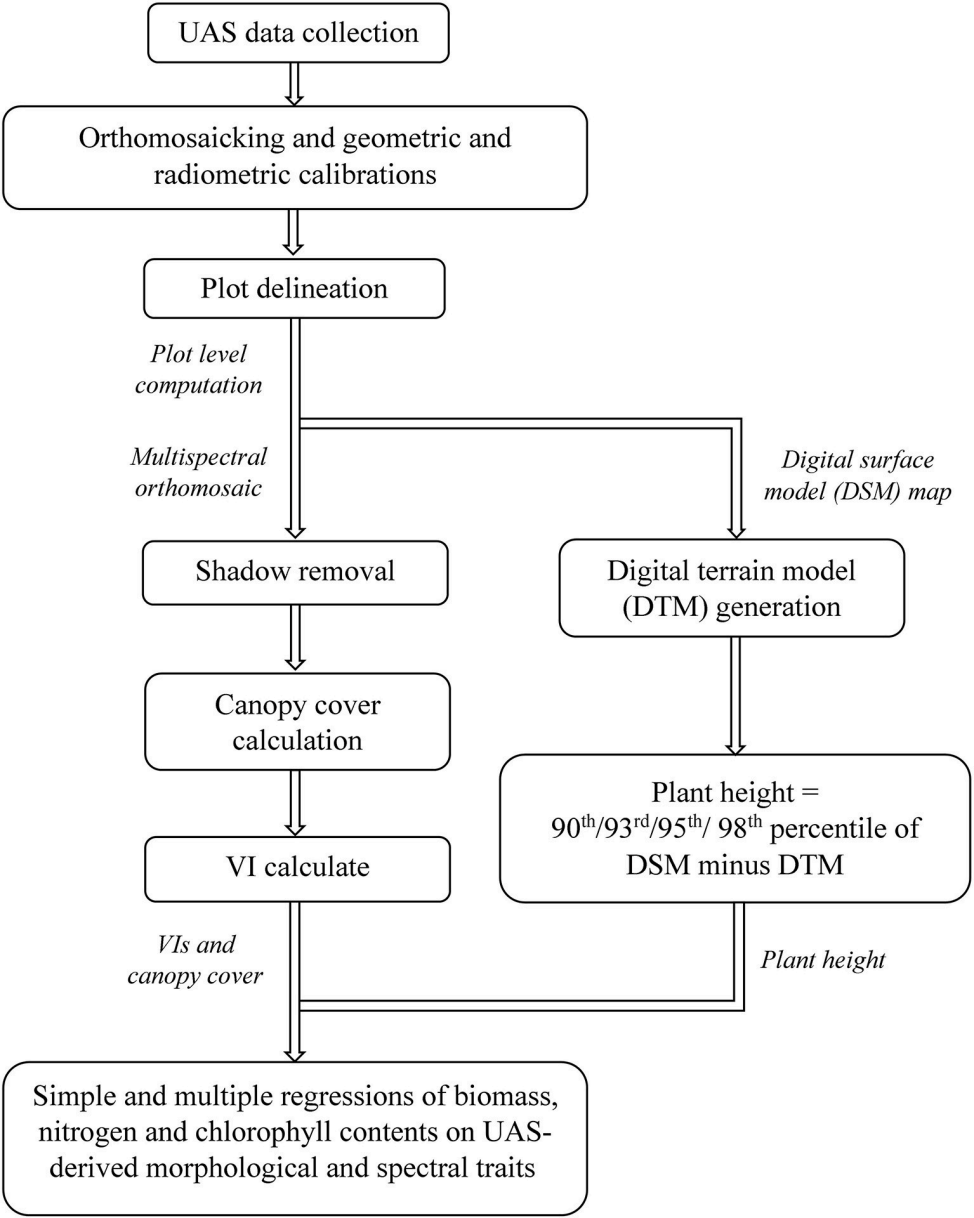
Flowchart of main processing steps in this study.

A flowchart has been provided in Figure 1, summarizing the main steps of this study: image data collection, image pre-processing, morphological and spectral trait extraction, and statistical analysis.

### Field experimental design

**Figure 2:**
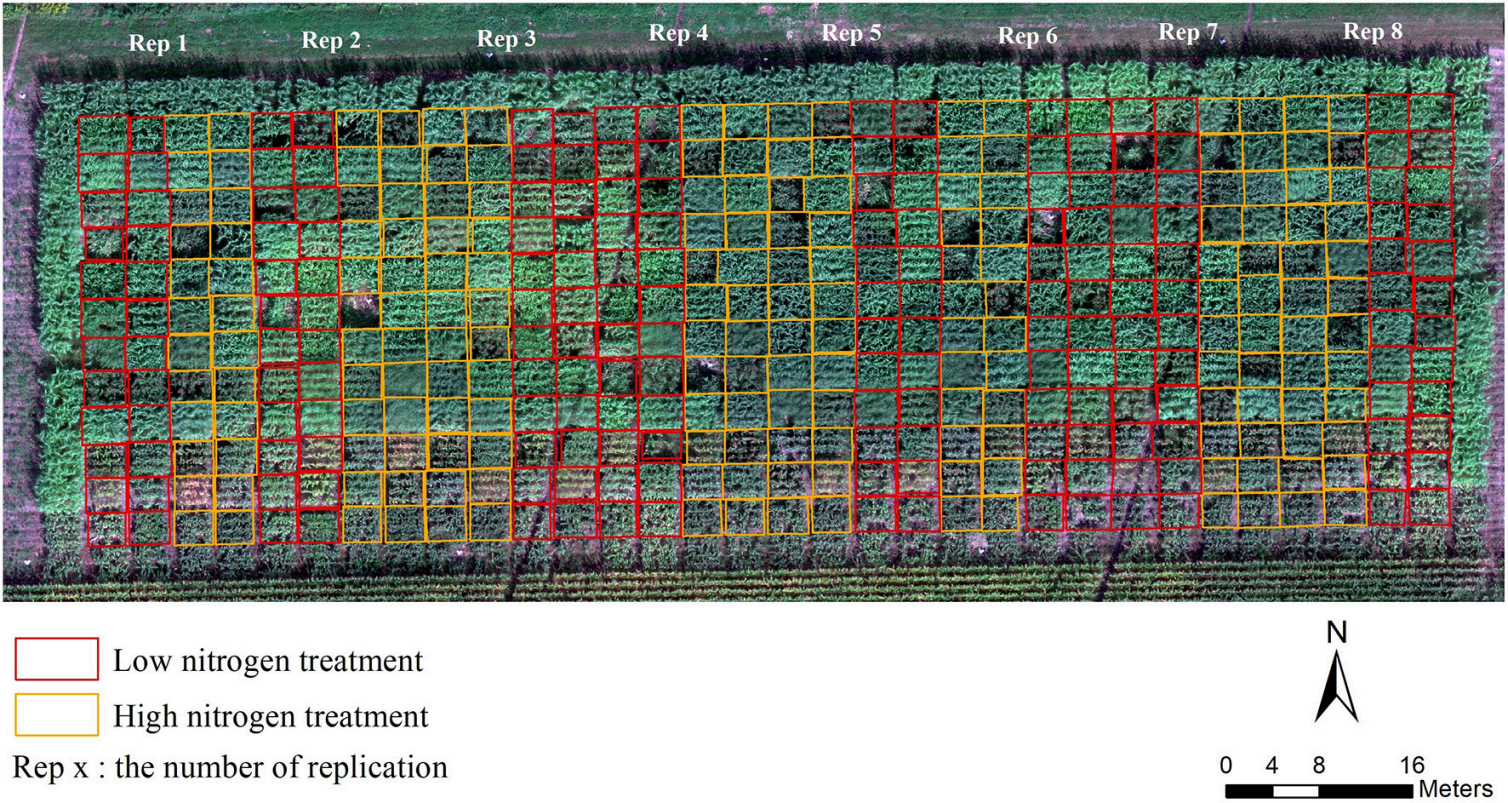
True-color orthomosaic showing field design. Images collected on September 11, 2017.

Field location and plot design—The field experiment was conducted over a 1.38 ha sorghum nitrogen stress trial located in Central City, Nebraska, US (41°12′3.0″ N, 97°56′40.56″W), in the growing season of 2017. The field was planted on May 26, 2017 with 24 sorghum lines (see Supplementary Table 1) in two nitrogen treatments (for the low nitrogen treatment no nitrogen was applied and for the high treatment 85 pounds of nitrogen per acre were added) and eight replications in a randomized complete block design (Figure 2). Each plot was 3 m by 3 m containing four rows with 0.10 m within-row spacing and 0.76 m row spacing delineated by a rectangular in Figure 2. This field trail was located on a commercial farm with center pivot irrigation. Nine inches of irrigation was added contain 0.9 ppm nitrate.

Collection of hyperspectral radiometer data-The most recently fully expanded leaf was taken from two random plants within a plot. The ASD FieldSpec 4 Standard Res (Analytical Spectral Devices, Colorado) was then used to measure hyperspectral reflectance readings in the 350 to 2,500 nm wavelength range of the leaf. The methods used here for the ASD readings are described in Yendrek et al. (2017). Both ends of the leaf were removed leaving roughly six to eight inches of the mid-section of the leaf. The leaf tissue on both sides of the midrib were removed from the midrib. One side was put into a paper envelope and dried at 50°C for nitrogen analysis and the other was placed into an aluminum foil packet which was then put on dry ice and subsequently stored at −80°C.

Laboratory analysis of leaf tissue—Frozen leaf issue was removed from −80°C and placed on dry ice. In a darkened room leaf disks (6 mm diameter) were punched from frozen leaves in the weigh boat, on dry ice until there was approximately 90 mg of leaf tissue which was about 18 to 27 disks. Prior to extraction 2.5 mL of 100% methanol was added to 15 mL tubes. Three replicates containing 30 mg or six to nine disks were taken and submerged in the methanol. The tubes were then placed in a rack in the dark and placed on a rotary shaker at 250 RPM for 24 h. After 24 h 200 mL of each sample was used to fill a 96 well black sided plate (Corning™ Costar) with a clear flat bottom, with one of the wells being filled with 200 μL of 100% methanol to be used as the blank. The top of the plate is secured with sealing film to prevent methanol evaporation. The plate was then read three times at 666 nm for chlorophyll A, 653 nm for chlorophyll B, 470 nm for carotenoids using a BioTek Synergy H1 Hybrid Reader. The chlorophyll/methanol equation (Lichtenthaler and Wellburn, 1983) was then used in to calculate chlorophyll A, chlorophyll B, and carotenoids of the extracts. The average of the three replicates was calculated for each sample. For nitrogen analysis the leaves were roughly chopped with a stainless-steel scissors in the envelopes and then sent to Ward Labs (Kearney, NE) for analysis of total nitrogen.

Field ground truth measurements—Plant height was measured on September 7, 2017 and October 9, 2017 from all eight replicates for each treatment. Plant heights were measured as the average height of plants in one of the center rows of the 4-row plot. It was measured on plants in the middle of the 3-meter row with a telescoping measuring stick which allows you to look up to align the top of the stick with top of the plants then record the height at eye level. Total above ground biomass was harvested on October 9, 2017 at which time fresh and dry above ground biomass (leaf and stem) were sampled in 363 plots. Eight replicates from each treatment were measured. A 0.91 m section of row in the middle of the plots was identified and plants were cut down at the bottom of the stem at soil level. Stalks with leaves and panicles were weighed and recorded separately on scales in the field but only the weight of stalks with leaves were used as the fresh biomass in this study. Subsamples of three stalks with leaves were reweighed to get the fresh weight and then bagged, oven dried and used to calculate the dry to fresh weight ratios which was then used to calculate the dry weights of the plots.

### UAS, sensors and flights

The system used for image capture was a Matrice 600 Pro multi-rotor UAS platform (DJI, Shenzhen, China), equipped with a Zenmuse X3 RGB camera (DJI, Shenzhen, China) and a multispectral camera RedEdge (MicaSense, Seattle, UAS). The RGB camera has 4000 by 2250 effective pixels. The multispectral camera system has five spectral bands blue, green, red, red edge, and near infrared (Table 1), each with 1280 by 960 effective pixels, and a downwelling light sensor system installed horizontally on top of the UAS used to measure the environmental irradiance and post-calibrate reflectance readings. As another source of radiometric calibration data, a standard calibration panel came with the multispectral camera was imaged on the ground before or after each flight.

**Table 1:** Center wavelength and full width at half maximum (FWHM) bandwidth of each spectral band of the RedEdge multispectral camera.

**Spectral band**	**Center wavelength (nm)**	**Bandwidth FWHM (nm)**
Blue	475	20
Green	560	20
Red	668	10
Red edge	717	10
Near infrared	840	40

Three flights were conducted on July 17, August 19, and September 11 in 2017. Flights were auto-piloted using DJI GO and DJI GS Pro applications with 92% forward and side overlap between images at 30 m above ground level. The resulting ground sampling distance (GSD) was 1.3 cm/pixel for RGB image and 2.0 cm/pixel for the multispectral image. The flight altitude and image acquisition parameters were tested and determined to optimize the flight duration and the quality of mosaicked maps. Eleven ground control points (GCPs) were distributed along the edges and inside the field each time before the flight for geometric calibration in image processing. Their geo-coordinates were accurately measured by a survey-grade RTK-GPS with less than 3 cm level accuracy.

### Image pre-processing

Two main tasks were completed in the pre-processing stage: the orthomosaic map and digital surface model (DSM) generation with proper geometric and radiometric calibrations; and the plot delineation to prepare for later processing.

RGB images were mosaicked by Pix4Dmapper software (Pix4D, Lausanne, Switzerland). Basically, there were three steps in Pix4Dmapper: Initial Processing; Point Cloud and Mesh; DSM, Orthomosaic and Index. In the initial processing, RGB raw images were imported into to extract and match key-points among neighboring images to form a rough mosaic. Geo-coordinates of the centers of GCPs were imported for geometric calibration and improving the initial mosaicking to form 3D point cloud and mesh. The final outputs were the 2D orthomosaic and DSM.

Multispectral images were mosaicked by Atlas Cloud service (MicaSense, Seattle, USA). Radiometric calibration was automatically addressed during this process using the irradiance measured in the field from the standard calibration panel and downwelling light sensor. The five-layer, 16-bit GeoTIFF output from Atlas was converted to five-layer reflectance GeoTIFF following the sensor instruction with a pixel value of 32768 equal to 100% reflectance. The multi-layer reflectance orthomosaic generated from the multispectral images were used later to estimate canopy cover and calculated various vegetation indices.

In order to conduct plot-based analysis, each plot boundary was delineated in the multispectral orthomosaic and DSM maps with unique plot ID as shapefiles in ArcGIS (Figure 2). The shapefiles, DSMs and multispectral orthomosaics were exported to R software for further data analysis.

**Figure 3:**
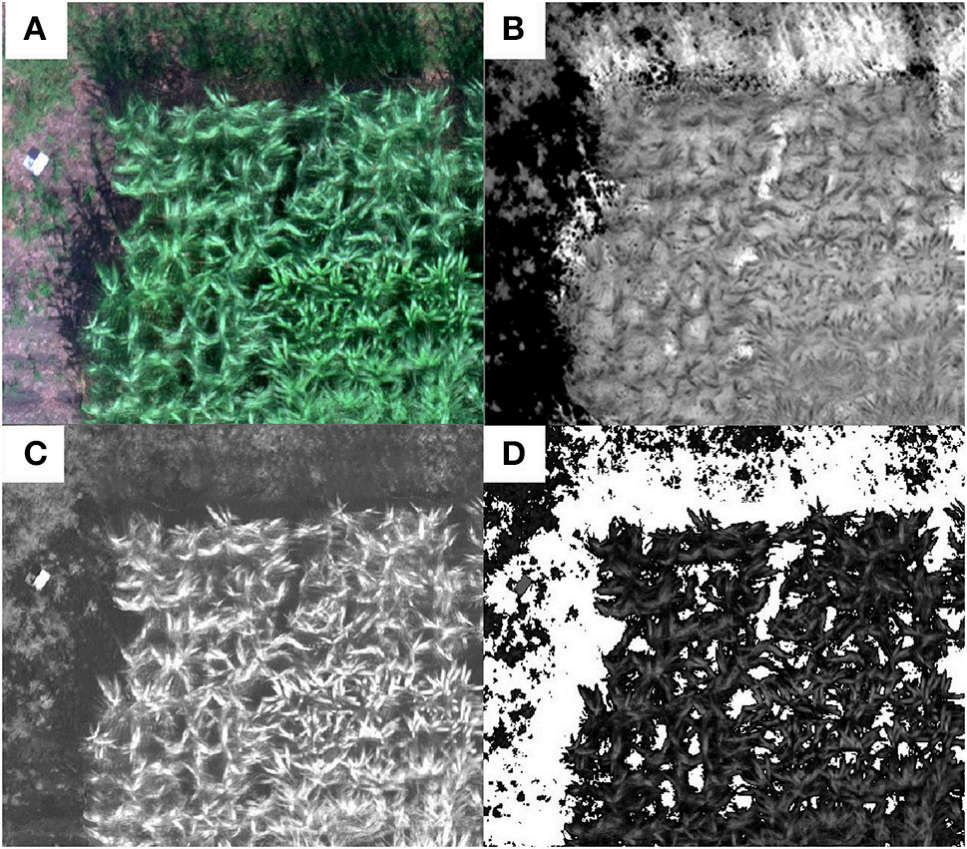
Shadow removal and vegetation segmentation from soil background using an example plot: **(A)** the RGB composite of the multispectral mosaic, **(B)** NDVI map showing the shaded area had higher NDVI values than canopy pixels, **(C)** excess green (ExG) image in which both the soil and shaded pixels have lower values than the sunlit vegetation pixels, and **(D)** mask for vegetation pixels (white pixels are soil and shaded pixels).

Shaded area would affect the reflectance recorded by camera, which was more pronounced when the plant was bigger. For the data set collected on the last date when sorghum plants were at their maximum height, more shadows were cast over neighboring shorter vegetation rows and soil. This was noticed in the multispectral images where shadowed vegetation and soil pixels had abnormally higher VI values than the sunlit vegetation pixels (Figure 3B) which was also observed in previous studies (Woebbecke et al., 1995). Those pixels with abnormal VI values were filtered out in this study and only sunlit vegetation pixels were used for VI calculation in each plot. To eliminate the shadow interference on plant VI calculation, the ExG index (Table 2) map, which was applied in other studies in distinguishing vegetative areas from soil or residue background (Woebbecke et al., 1995), was calculated. In this study, both the soil and shaded vegetation pixels in the ExG index map had lower values than the sunlit vegetation pixels (Figure 3C) so that they were filtered out and only the sunlit vegetation pixels were retained for VI calculation. A threshold of 0.046 was determined by trial and error and used to segment the vegetation pixels from the soil pixels in the ExG map (Figure 3D). The segmented vegetation pixels formed a mask which was applied in the further processing for ground cover estimate and VI calculations.

**Table 2:** Formulas of vegetation indices used in this study.

**VIs**	**Formula**	**Feature/application**
ExG	2 × ρ_*Green*_−ρ_*Red*_−ρ_*Blue*_	Distinguishes vegetation from soil background
NDVI	(ρ_*NIR*_−ρ_*Red*_)/(ρ_*NIR*_+ρ_*Red*_)	Correlates with green biomass, chlorophyll
RDVI	$\left(\rho_{NIR}-\rho_{Red}\right)/\sqrt{\left(\rho_{NIR}+\rho_{Red}\right)}$	Less sensitive to the interfering effects of soil
GNDVI	(ρ_*NIR*_−ρ_*Green*_)/(ρ_*NIR*_+ρ_*Green*_)	Correlates with *Chlorophyll-a*
CI_Green_	(ρ_*NIR*_/ρ_*Green*_)−1	Correlates with chlorophyll and nitrogen
CI_RedEdge_	(ρ_*NIR*_/ρ_*RedEdge*_)−1	Correlates with chlorophyll and nitrogen
NDRE	(ρ_*NIR*_−ρ_*RedEdge*_)/(ρ_*NIR*_+ρ_*RedEdge*_)	Correlates with chlorophyll or nitrogen
RGBVI	$\left({\rho_{Green}}^{2}-\rho_{Blue}\times \rho_{Red}\right)/\left({\rho_{Green}}^{2}+\rho_{Blue}\times \rho_{Red}\right)$	Estimates biomass

*ρ means spectral reflectance.

### Morphological and spectral traits extraction

Morphological and spectral plant traits were extracted from the pre-processed data to estimate sorghum biomass and nitrogen and chlorophyll contents, including plant height, canopy cover, and various VIs at the individual plot level.

Plant height was derived by subtracting the digital terrain model (DTM) from the digital surface model (DSM). The DSM was generated along with the orthomosaic from mosaicking the RGB images in Pix4D, and was geometrically calibrated with the GCP's coordinates surveyed by the RTK-GPS during field data collections. The DTM was generated by linearly interpolating the soil surface on east and west side of the field, assuming little elevation changes within this 1.38 ha field. The derived plant height map had the same spatial resolution of 1.3 cm as the RGB orthomosaic. The 90th, 93rd, 95th, and 98th percentiles of all pixels falling into a plot boundary were calculated and compared with the manually sampled plant height in the same plot to find the one with highest correlation. After shadow removal, canopy cover was calculated as the ratio of the number of segmented sunlit vegetation pixels to the total number of pixels in a plot (Lee and Lee, 2011).

Calibrated reflectance in each multispectral band was extracted, and various VIs were calculated for each plot by averaging the VI values of all pixels of interest within the plot boundary. These VIs included normalized difference vegetation index (NDVI), renormalized difference vegetation index (RDVI) (Roujean and Breon, 1995), green normalized difference vegetation index (GNDVI) (Gitelson et al., 1996), green chlorophyll index (CI_Green_) and red edge chlorophyll index (CI_RedEdge_) (Schlemmera et al., 2013), normalized difference red edge index (NDRE) (Fitzgerald et al., 2006), and RGB vegetation index (RGBVI) (Bendig et al., 2015) (Table 2). NDVI is one of the most commonly used indices for estimating crop physiological traits such as chlorophyll. RDVI uses the same spectral bands as NDVI; however, RDVI is less sensitive to the variation of soil background. Given the saturation problem of NDVI after canopy closure, RDVI may be considered superior (Fu et al., 2013). GNDVI was found to have wider dynamic range than NDVI and is more sensitive to *chlorophyll-a* concentration (Gitelson et al., 1996). Similar to NDVI, NDRE is a good indicator of chlorophyll or nitrogen status (Fitzgerald et al., 2006); however, the replacement of red band with the red edge band makes NDRE more sensitive to the biomass change than NDVI after canopy closure. Using bands in the visible spectra, RGBVI can be used to estimate biomass (Bendig et al., 2015).

### Statistical modeling for biomass, nitrogen, and chlorophyll contents

Since the biomass was sampled late in the season close to the last flight date, only the UAS data collected on September 11, 2017 was used for the biomass analysis. To estimate the fresh and dry biomass using remotely sensed plant traits, simple exponential regression (SER) models were first built using univariate morphological or spectral trait. Given that the biomass is intuitively related to multiple traits such as plant height, and stalk diameter, it is also worth investigating the integration of more than one trait using multiple exponential regression (MER) models to see if the estimation of biomass can be improved. To select predictors for the MER models, a correlation matrix was first calculated to avoid including predictors that were highly correlated (Table 3). In this study, Pearson's correlation coefficient r was used and the strength of the correlation was determined as summarized by Asuero et al. (2006): r ranging from 0 to 0.29 was interpreted as little if any correlation, r ranging from 0.30 to 0.49 was regarded as low correlation, and r ranging from 0.50 to 0.69 was moderate correlation, while r greater than 0.69 was high to very high correlation. In this study, paired predictors with r lower than 0.69 were selected to be included in the regression models. Based on that, the following ten combinations of predictors were investigated: plant height and canopy cover, NDVI and RGBVI, NDRE and RGBVI, RDVI and RGBVI, plant height and NDRE, plant height and RGBVI, canopy cover and NDVI, canopy cover and NDRE, canopy cover and RDVI, plant height and canopy cover and NDRE.

**Table 3:** Correlation matrix of candidate predictors in biomass prediction.

	**Plant height**	**Canop cover**	**NDVI**	**NDRE**	**RDVI**	**RGBVI**
Plant height	1					
Canopy cover	0.61	1				
NDVI	0.74	0.54	1			
NDRE	0.68	0.25	0.90	1		
RDVI	0.80	0.57	0.96	0.89	1	
RGBVI	0.54	0.75	0.64	0.26	0.59	1

*r ranging from 0 to 0.29 was interpreted as little if any correlation (highlighted in blue), r ranging from 0.30 to 0.49 was regarded as low correlation, and r ranging from 0.50 to 0.69 was moderate correlation (highlighted in yellow), and r greater than 0.69 was high to very high correlation*.

Three hundred and Sixty-Three samples from the September 11 flight were divided into training set (290 samples) and testing set (73 samples) in a ratio of 4:1. The training set was used to build regression models which were validated using 10-fold cross validation. The validation results were reported using averaged root mean square error (*RMSE*) of the 10 folds (Equation 1) and standard deviation (*STD*) (Equation 2) of 10 *RMSE* values derived from the 10-fold cross validation. The established regression models were further tested using the testing set, and were evaluated using the Pearson correlation coefficients (*r*) (Equation 3) and *RMSE*.

(1) $$\begin{array}{l} RMSE=\sqrt{\frac{1}{n}\sum_{i=1}^{n}{\left(P_{i}-M_{i}\right)}^{2}} \end{array}$$

(2) $$\begin{array}{l} STD=\sqrt{\frac{\sum_{i=1}^{n}{\left(x_{i}-\bar{x}\right)}^{2}}{n-1}} \end{array}$$

(3) $$\begin{array}{l} r=\frac{\sum_{i}^{n}\left(M_{i}-\bar{M}\right)\left(P_{i}-\bar{P}\right)}{\sqrt{\sum_{i}^{n}{\left(M_{i}-\bar{M}\right)}^{2}}\sqrt{\sum_{i}^{n}{\left(P_{i}-\bar{P}\right)}^{2}}} \end{array}$$

where *n* is the number of samples, *P*_*i*_ stands for predicted value, *M*_*i*_ stands for manually measured value, $\bar{P}$ is the mean of predicted values, $\bar{M}$ is the mean of manually measured values. *x*_i_ is the observed values, and $\bar{x}$ is the mean value of these observations.

In order to examine the effect of nitrogen treatments, *t*-tests were conducted using three VIs (CI_Green_, CI_RedEdge_, and NDRE) calculated from the last flight (September 11, 2017) between high nitrogen (192 plots) and low nitrogen (192 plots) treatments.

Furthermore, to evaluate the relationship between various VIs and sorghum chlorophyll and nitrogen contents, *r* was calculated between the various VIs and the manually measured leaf chlorophyll and nitrogen contents for sampled sorghum plants from each plot. For chlorophyll, 70 plots in July, 68 plots in August, and 112 plots in September had valid samples (250 samples in total); for nitrogen, 50 plots in July, 50 plots in August, and 69 plots in September had valid samples (169 samples in total).

## Results

### Plant height estimation

**Figure 4:**
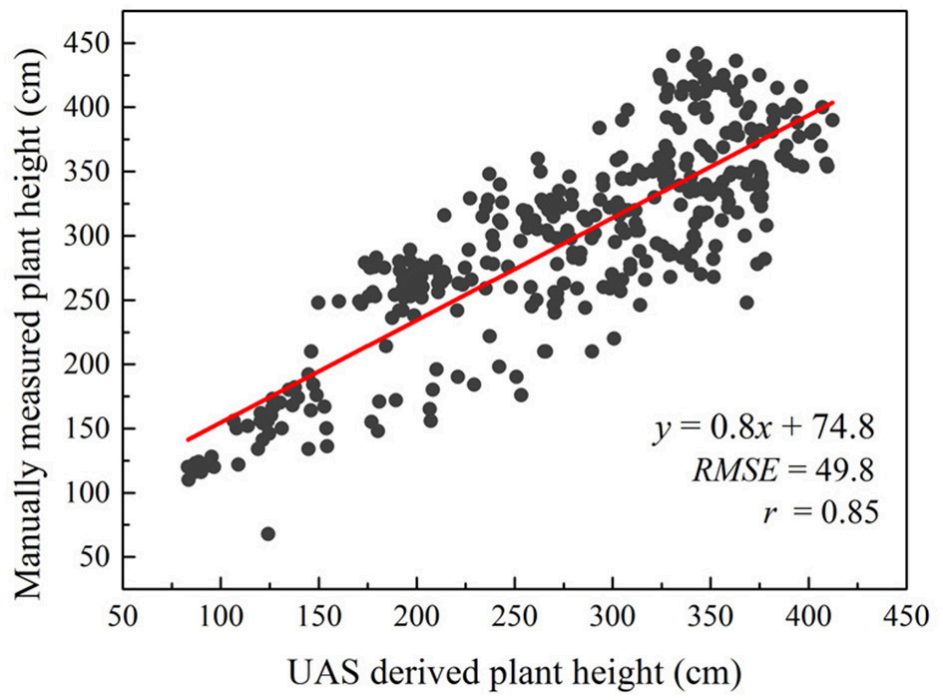
Correlation between UAS estimated plant height and manually measured plant height over 363 plots on September 11, 2017.

Compared to other percentile values, a stronger linear correlation was obtained (*r* = 0.85) between the 90th percentile of estimated plant height from the RGB orthomosaic and the manually sampled plant height in 363 plots in September 2017 (Figure 4). The *RMSE* was 49.8 cm and the *r* was 0.85 between UAS derived plant height and manually measured plant height. The coefficient of variation (CV) for aerial data estimated plant height was 32.11% and for manually measured plant height was 27.92%.

### Fresh and dry biomass estimation

**Figure 5:**
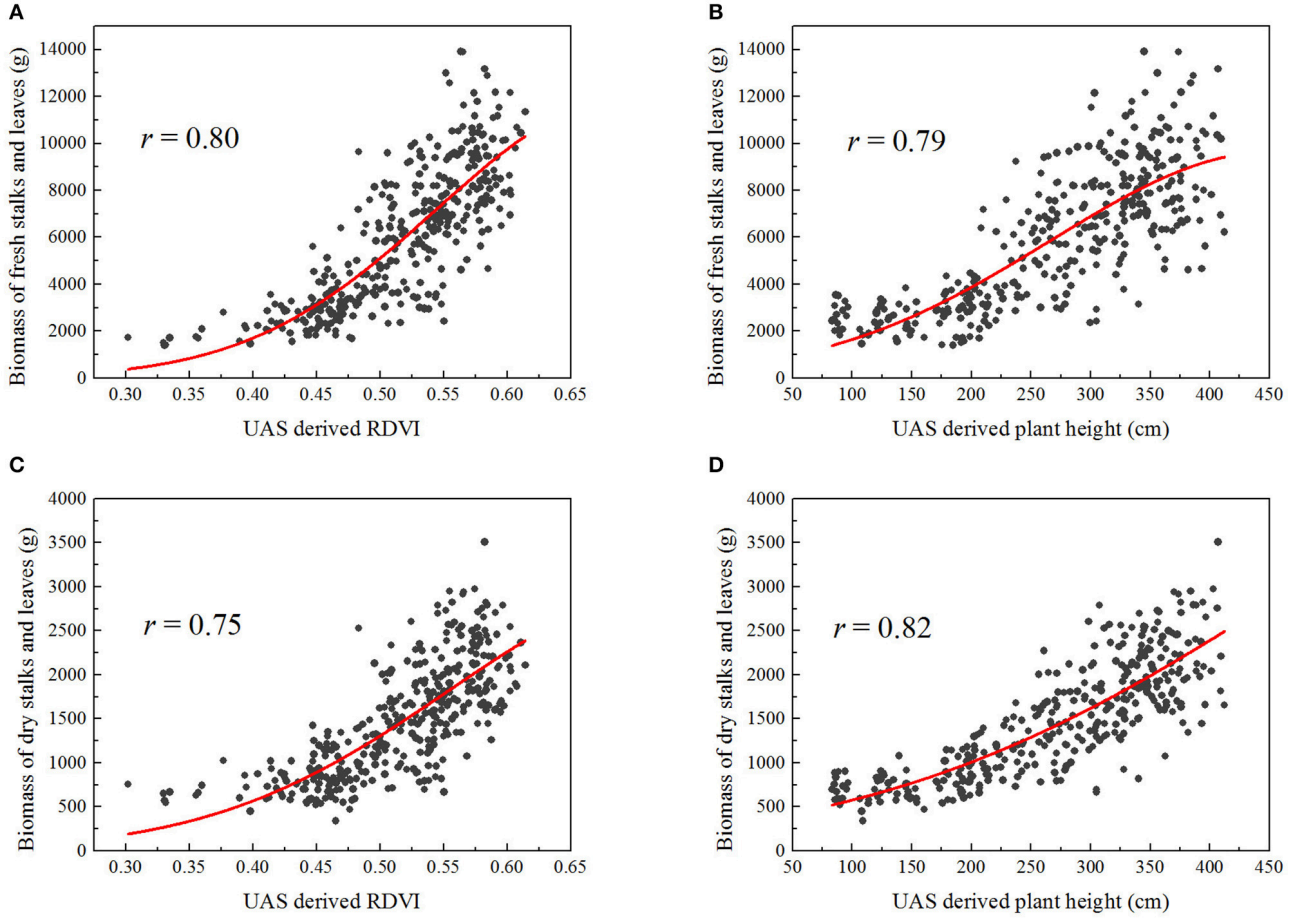
Exponential correlations between some UAS-derived traits and the manually sampled biomass of stalks and leaves (fresh or dry), over 363 plots: **(A)** RDVI and fresh biomass, **(B)** plant height and fresh biomass, **(C)** RDVI and dry biomass, **(D)** plant height and dry biomass.

The correlations between a single UAS-derived morphological or spectral trait and the manually sampled fresh or dry biomass tended to be exponential rather than linear in this study (Figure 5).

For the simple exponential model of fresh biomass (Table 4), plant height gave higher correlation than canopy cover (*r* = 0.81), whereas RDVI and NDVI provided better results than the other VIs (*r* = 0.83 for RDVI, and *r* = 0.80 for NDVI).

**Table 4:** Fresh biomass estimation results in 10-fold cross validation as well as in the testing set, based on simple exponential regression (SER) and multiple exponential regression (MER) models.

**Model**	**Predictors**	**Training set: average of 10-fold cross validation**		Formula^*^	**Testing set**	
		***RMSE* (kg)**	***STD* (kg)**		***RMSE* (kg)**	***r***
SER	Plant height	23.78	4.06	*Y* = 21.22 × *e*^(0.005 × *PH*)^	26.25	0.81
	Canopy cover	32.30	5.16	*Y* = 22.47 × *e*^(1.618 × *CC*)^	35.78	0.62
	NDVI	23.67	3.11	*Y* = 0.31 × *e*^(7.03 × *NDVI*)^	25.89	0.80
	NDRE	28.19	3.40	*Y* = 14.02 × *e*^(3.813 × *NDRE*)^	31.88	0.66
	RDVI	18.10	3.60	*Y* = 2.04 × *e*^(6.973 × *RDVI*)^	24.26	0.83
	RGBVI	32.73	4.69	*Y* = 6.41 × *e*^(3.68 × *RGBVI*)^	36.79	0.57
MER	Plant height and canopy cover	23.73	4.10	*Y* = 18.70 × *e*^(0.004 × *PH*+0.246 × *CC*)^	25.98	0.81
	NDVI and RGBVI	23.69	3.11	*Y* = 0.26 × *e*^(6.637 × *NDVI*+0.706 × *RGBVI*)^	25.62	0.80
	NDRE and RGBVI	23.89	3.57	*Y* = 1.36 × *e*^(3.605 × *NDRE*+3.571 × *RGBVI*)^	24.92	0.82
	RDVI and RGBVI	23.25	3.54	*Y* = 1.32 × *e*^(6.41 × *RDVI*+1.069 × *RGBVI*)^	23.70	0.84
	Plant height and NDRE	23.32	3.81	*Y* = 16.54 × *e*^(0.004 × *PH*+1.166 × *NDRE*)^	25.54	0.81
	Plant height and RGBVI	23.31	3.92	*Y* = 9.37 × *e*^(0.004 × *PH*+1.370 × *RGBVI*)^	25.14	0.83
	Canopy cover and NDVI	23.01	3.28	*Y* = 0.31 × *e*^(0.584 × *CC*+6.447 × *NDVI*)^	24.81	0.82
	Canopy cover and NDRE	24.35	3.89	*Y* = 5.86 × *e*^(1.336 × *CC*+3.486 × *NDRE*)^	25.76	0.80
	Canopy cover and RDVI	23.10	3.81	*Y* = 1.96 × *e*^(0.434 × *CC*+6.412 × *RDVI*)^	23.81	0.83
	Plant height, canopy cover, and NDRE	22.82	3.89	*Y* = 10.63 × *e*^(0.003 × *PH*+0.6 × *CC*+1.728 × *NDRE*)^	24.47	0.83

* *STD is standard deviation of 10 RMSE values of the total plot weight from the 10-fold cross validation; Y, predicted fresh biomass (kg/plot); PH, plant height; CC, canopy cover; NDVI, normalized difference vegetation index; NDRE, normalized difference red edge index; RDVI, renormalized vegetation index; and RGBVI is the RGB vegetation index*.

Slightly better correlations were obtained when multiple traits were combined into the fresh biomass regression model, with the outcome being that *r* was greater than 0.80 for all combinations. Interestingly, when used individually in the simple exponential models, either NDRE or RGBVI resulted in lower correlations (*r* = 0.66 for NDRE, *r* = 0.57 for RGBVI); however, the combination of them using the multiple exponential model largely improved the correlation with fresh biomass (*r* = 0.82). Similar results were found in prediction of dry biomass. The morphological trait plant height (*r* = 0.87), spectral traits RDVI (*r* = 0.78) and NDVI (*r* = 0.78) individually exhibited better correlations with the dry biomass using simple exponential models; while the combination of them with other traits did not significantly improve the results in this case (Table 5).

**Table 5:** Dry biomass estimation results in 10-fold cross validation as well as in the testing set, based on simple exponential regression (SER) and multiple exponential regression (MER) model.

**Model**	**Predictor(s)**	**Training set: average of 10-fold cross validation**		Formula^*^	**Testing set**	
		***RMSE* (kg)**	***STD (kg)***		***RMSE* (kg)**	***r***
SER	Plant height	4.83	0.70	*Y* = 5.63 × *e*^(0.004 × *PH*)^	4.89	0.87
	Canopy cover	7.20	0.93	*Y* = 6.50 × *e*^(1.42 × *CC*)^	7.89	0.59
	NDVI	5.73	0.67	*Y* = 0.22 × *e*^(5.679 × *NDVI*)^	5.92	0.78
	NDRE	6.34	0.78	*Y* = 4.46 × *e*^(3.272 × *NDRE*)^	6.70	0.70
	RDVI	5.58	0.69	*Y* = 0.91 × *e*^(5.869 × *RDVI*)^	5.83	0.78
	RGBVI	7.51	0.74	*Y* = 3.04 × *e*^(2.726 × *RGBVI*)^	8.41	0.48
MER	Plant height and canopy cover	4.82	0.70	*Y* = 5.15 × *e*^(0.004 × *PH*+0.169 × *CC*)^	4.88	0.87
	NDVI and RGBVI	5.74	0.67	*Y* = 0.22 × *e*^(5.607 × *NDVI*+0.130 × *RGBVI*)^	5.92	0.78
	NDRE and RGBVI	5.75	0.73	*Y* = 0.86 × *e*^(3.125 × *NDRE*+2.522 × *RGBVI*)^	5.74	0.79
	RDVI and RGBVI	5.58	0.69	*Y* = 0.77 × *e*^(5.664 × *RDVI*+0.392 × *RGBVI*)^	5.82	0.78
	Plant height and NDRE	4.81	0.68	*Y* = 5.03 × *e*^(0.004 × *PH*+0.545 × *NDRE*)^	4.78	0.87
	Plant height and RGBVI	4.82	0.68	*Y* = 4.47 × *e*^(0.004 × *PH*+0.384 × *RGBVI*)^	4.87	0.87
	Canopy cover and NDVI	5.59	0.53	*Y* = 0.22 × *e*^(0.61 × *CC*+5.085 × *NDVI*)^	5.71	0.79
	Canopy and NDRE	5.52	0.56	*Y* = 1.94 × *e*^(1.222 × *CC*+3.041 × *NDRE*)^	5.40	0.82
	Canopy cover and RDVI	5.49	0.69	*Y* = 0.86 × *e*^(0.472 × *CC*+5.278 × *RDVI*)^	5.74	0.78
	Plant height, canopy cover, and NDRE	4.76	0.67	*Y* = 3.90 × *e*^(0.003 × *PH*+0.345 × *CC*+0.87 × *NDRE*)^	4.68	0.88

* *STD is standard deviation of 10 RMSE values of the total plot weight from the 10-fold cross validation; Y, predicted dry biomass (kg/plot); PH, plant height; CC, canopy cover; NDVI, normalized difference vegetation index; NDRE, normalized difference red edge index; RDVI, renormalized vegetation index; and RGBVI is the RGB vegetation index*.

### Nitrogen treatment effect on UAS-derived traits

**Figure 6:**
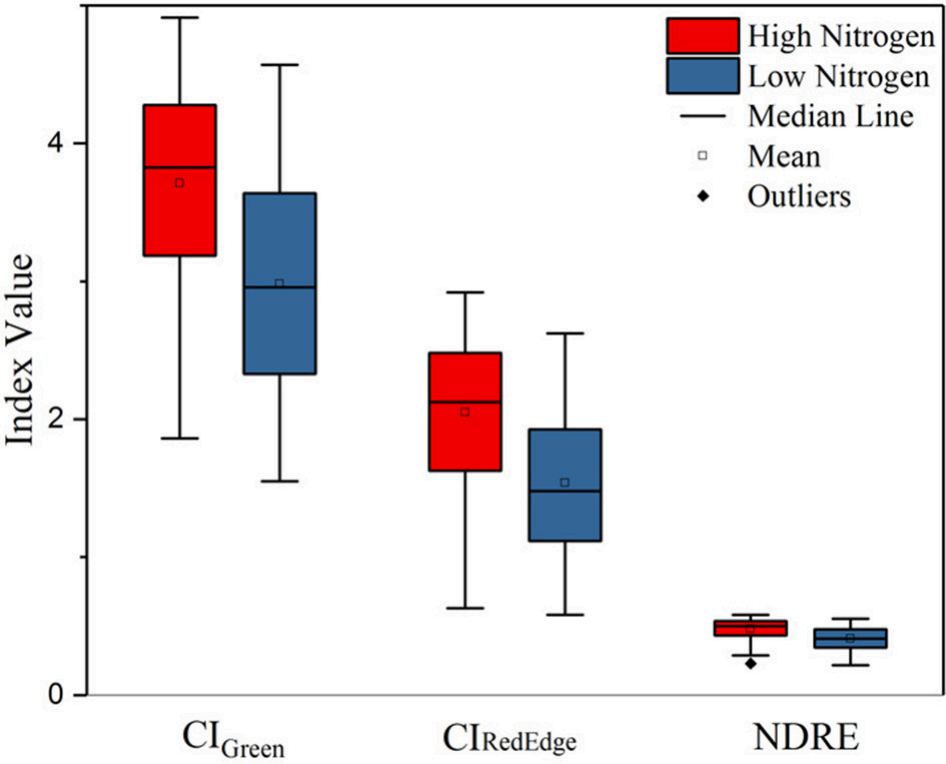
Boxplot of three VIs (CI_Green_, CI_RedEdge_, and NDRE) derived from the late season multispectral images of the low and high nitrogen treatments.

As shown in Table 6, significant differences were found in the remotely sensed three VIs between high and low nitrogen treatments (*p* < 0.0001). It is shown in Figure 6 that the nitrogen effect can be clearly distinguished with the three selected VIs—CI_Green_, CI_RedEdge_, and NDRE—derived from the late season UAS data.

**Table 6:** Student's *t*-test results showing significant differences of remotely sensed VIs between low (192 plots) and high (192 plots) nitrogen treatments.

**VI**	***t***	***p*-value**
CI_Green_	9.8025	<2.2e−16
CI_RedEdge_	9.5994	<2.2e−16
NDRE	9.1623	<2.2e−16

### Chlorophyll and nitrogen content estimation

Moderate to strong correlations (*r* > 0.5) were found between the chlorophyll content of leaf samples and the corresponding VIs, except NDVI, of same plots calculated from multispectral aerial data over July, August, and September in 2017 (Table 6). Similar results were found for the nitrogen content (Table 7). These VIs included CI_Green_, CI_RedEdge_, GNDVI, and NDRE. VIs were also calculated from the leaf-level hyperspectral measurements sampled in the same plots. Similar correlations (*r* > 0.4) were found when the specific spectral bands were taken from the hyperspectral radiometer data as those calculated from the multispectral aerial data (Table 7). Although correlation using NDVI improved with the hyperspectral radiometer, the NDVI index had lower correlations with chlorophyll and nitrogen contents than other VIs, while NDRE remained the VI with highest correlation with nitrogen and chlorophyll in this case.

**Table 7:** Pearson correlation coefficients between the chlorophyll and nitrogen contents of leaf samples, and the corresponding VIs of same plots calculated from multispectral aerial data and leaf-level hyperspectral measurements, using data set collected over three flights in 2017.

**Sensor**	**Traits**	**Chlorophyll**		**Nitrogen %**	
		***r***	***p*-value**	***r***	***p*-value**
MicaSense RedEdge® multispectral camera (on UAS)	CI_green_	0.53^***^	<2.2e−16	0.55^***^	6.65e−15
	CI_RedEdge_	0.53^***^	<2.2e−16	0.58^***^	<2.2e−16
	GNDVI	0.55^***^	<2.2e−16	0.58^***^	2.588e−16
	NDVI	0.17^**^	0.0088	0.31^***^	3.736e−5
	NDRE	0.55^***^	<2.2e−16	0.61^***^	<2.2e−16
ASD FieldSpec® hyperspectral sensor with leaf clip (only the same spectral bands as RedEdge® were used)	CI_green_	0.42^***^	6.733e−12	0.55^***^	6.899e−15
	CI_redEdge_	0.50^***^	<2.2e−16	0.60^***^	<2.2e−16
	GNDVI	0.44^***^	3.247e−13	0.58^***^	<2.2e−16
	NDVI	0.27^***^	1.731e−5	0.42^***^	1.014e−8
	NDRE	0.51^***^	<2.2e−16	0.62^***^	<2.2e−16

*** *Correlation is significant at the 0.001 level*.

** *Correlation is significant at the 0.01 level*.

## Discussion

The moderate to strong correlations (*r* varied from 0.55 to 0.88) found between the UAS-derived plant morphological and spectral traits and the sorghum late-season biomass, nitrogen, and chlorophyll contents in this study indicates that UAS should be useful for phenotyping. Compared with the hyperspectral reflectance that was manually sampled at the leaf level using hyperspectral radiometer with a leaf clip, the UAS-derived VIs using the five-band multispectral camera resulted in similar correlations with nitrogen and chlorophyll contents when the same VIs were calculated from the measured hyperspectral reflectance (Table 7). This not only demonstrates the fidelity of the UAS-based remote spectral sensing, but also indicates the potential for scaling up the high-throughput phenotyping from ground-based leaf level to UAS-based canopy level assessment.

When a single trait was used for prediction with simple exponential regression models, estimated plant height, RDVI, and NDVI indices individually had the strongest correlations with both fresh and dry sorghum biomass among the various remotely sensed traits. The high and robust correlation derived from plant height was also found in a previous study in barley (Bendig et al., 2015). Interestingly, the NDRE index showed a little lower correlation to fresh and dry biomass (*r* within 0.66 and 0.70) than NDVI and NDRE but significantly outperformed NDVI in chlorophyll and nitrogen content estimations (Table 7) which showed NDRE's known advantage over NDVI after canopy closure due to the saturation in the red spectral band at the mid to late growth stages (Mutanga and Skidmore, 2004). Late season canopy cover had moderate correlations with biomass (*r* within 0.59 and 0.62) but was not shown to be superior to vegetation indices in our study. The inferior correlation of RGBVI index with biomass compared with other traits was also reported in barley (Tilly et al., 2015). However, it is noteworthy that, if no near-infrared spectral data and only the RGB information was available, the UAS-derived RGBVI index alone still provided low to moderate correlations with fresh and dry biomass (*r* within 0.48 and 0.57).

When several traits with multiple exponential regression models were used, similar correlations were achieved for fresh and dry biomass prediction as compared to the results derived from single traits in this study. The correlation using several traits was best when using the traits that had the strongest correlations individually, i.e., the plant height and RDVI indices in this case. Similar data fusion models that were investigated in previous studies varied in the ability to predict biomass over the single metric predictions and this depended on the traits that were added at different growth stages and the correlations between the traits. The integration of RGBVI and plant height resulted in small improvement in the biomass prediction in barley (*r* from 0.89 to 0.92) but no improvement in the biomass prediction was found with the integration of plant height and other VIs derived from visible and near-infrared spectra (Bendig et al., 2015). This can probably be explained by the moderate to high correlations between the plant height and various VIs found in this study (Table 3).

Further work will be needed to improve biomass prediction through the inclusion of additional morphological traits such as stalk diameter and additional or customized spectral bands. Some other traits that were not included in this study also showed ability to increase the estimation accuracy when combined with some of the UAS-derived traits in this study. When adding manually measured stem diameter on the UAS-derived plant height, the biomass prediction using a volumetric cylinder equation in corn was significantly improved (*r* from 0.56 to 0.93) (Varela et al., 2017). However, automating stem diameter measurements is challenging and may not be useful in many energy sorghum varieties that do not flower in North America (SD Kresovich, personal communication). Another case would be the combination of hyperspectral canopy reflectance and plant height which improved the accuracy of estimating winter wheat biomass (*r* from 0.73 to 0.91) (Yue et al., 2017). In addition, customization of spectral bands of the UAS-based multispectral sensor based on the feature spectral bands derived from the leaf-level or ground-based hyperspectral sensing (Yendrek et al., 2017) for specific applications can scale up the throughput of phenotyping capabilities in the field while reducing the sensor instrument cost.

Improvement can also be achieved by including temporal data during the growing season and using more sophisticated statistical models. The late-season biomass predicted by single or multiple UAS-derived traits had strong exponential correlations with the sampled fresh biomass (maximum *r* = 0.84) and dry biomass (maximum *r* = 0.88); however, no significant improvement was found if multiple traits collected on the same date were used to build models (Tables 4, 5). Similar results were reported in study on sorghum biomass prediction (Zhang et al., 2017) showing that more data on additional traits measured on the same day provided no significant improvement for biomass prediction. However, significant improvements were found when measurements from multiple time points with either a single trait or multiple traits were used (Zhang et al., 2017). Also, the exponential relationships found between UAS-derived traits and biomass in this study were similar to a previous finding in barley (Bendig et al., 2014).

We recommend segmenting vegetation pixels and shaded soil pixels for some VI calculations to avoid the interference of shaded soil pixels in the calculations. Shadows that were cast on the canopy and soil were identified in this study to have much higher NDVI values than the sunlit vegetation pixels (Figure 3). If an averaged VI value was calculated for all pixels encompassed within a plot boundary, plots with more shaded vegetation and soil areas may result with higher VI values than those without much shaded areas even though the NDVI of the actual leaves may be lower. Shading may not be a problem for production agricultural applications when the whole field was planted with same variety and population; however, in the application of phenotyping when many small plots that contained different varieties or uneven stands this cause substantial errors in estimating the true canopy VIs. Accurate segmentation of sunlit vegetation and shaded vegetation and soil pixels in the image processing is important to ensure the reliability of VI values. In this study, we used the ExG index map for segmentation which was effective but still resulted with some mis-classification of shaded vegetation and shaded soil pixels. This also resulted in a lower estimation of canopy cover especially for those plots with significant shadows. Future research will be needed to investigate the hyperspectral reflectance patterns of the sunlit and shaded vegetation and soil pixels and corresponding classification algorithms with proper band selection techniques (Sun et al., 2015) to customize multispectral cameras for high-throughput applications.

The results obtained using UAS-derived DSM to estimate plant height are very promising (*r* = 0.85) and are similar to other studies (Geipel et al., 2014; Chang et al., 2017b; Watanabe et al., 2017; Hu et al., 2018; Malambo et al., 2018; Pugh et al., 2018) Considering the strong correlation between the late-season plant height and the sampled fresh and dry biomass (*r* > = 0.81), this method can be used to quickly estimate plant biomass. The UAS-derived plant height in this study was only investigated using the data collected on a single date late in the season since the main purpose was end-of-season biomass prediction. The accuracy of the height estimation achieved in this study (*RMSE* = 49.8 cm) needs to be improved in order to be applied to plant height estimation in earlier growth stages when plants are smaller. Ideally, the structure from motion (SfM) algorithm used behind this technology to generate the point clouds or the structure of a targeted object can achieve reprojection error at only about one pixel (Snavely et al., 2008) which in our case would be about 1.3 cm accuracy. However, in real world agricultural applications, errors are induced due to the movement of the plant canopy by the wind (Chang et al., 2017b). During our data collections, the wind speed was 5 m/s (10 mph) which caused the top canopy to sway at decimeter level. This rendered the SfM algorithm difficult to use because matched keypoints among images taken from different angles were hard to find and therefore errors were generated. Moreover, variation in leaf angle, canopy structure and presence or absence of panicles among genotypes caused the discrepancy between manually sampled plant height and plant height estimated from the UAS-derived point clouds data. In addition, the accuracy of UAS-derived plant height also depends on the accuracy of the derived digital terrain model (DTM) or the elevation of the field (Malambo et al., 2018). In this study, we assumed a constant elevation change of the field since the field was flat and relatively small (1.38 ha); however, ignoring the within-field unevenness may induce a small amount of error. A pre-planting elevation mapping can largely reduce such errors.

Improving prediction accuracy to develop a more generally applicable model for energy sorghum will be a future goal. The models developed in this study were based on a single season and so they may be further improved through the incorporation of multi-season and multi environment data. Incorporating additional data sets such as growing degree days, precipitation, soil physicochemical properties, planting dates and other agronomic practices may all allow for the further improvement of predictive models using data from UAS.

## Author contributions

JL, A-NV-S, YS conducted the aerial data collections. DS provided the field, all the ground truth and hyperspectral radiometer data. JL and YS performed the aerial data analysis and wrote the manuscript. DS edited and reviewed the manuscript. All authors reviewed the manuscript and agreed with the submission.

### Conflict of interest statement

The authors declare that the research was conducted in the absence of any commercial or financial relationships that could be construed as a potential conflict of interest.
