# Supplementary material for: Elucidating Sorghum Biomass, Nitrogen and Chlorophyll Contents With Spectral and Morphological Traits Derived From Unmanned Aircraft System
Source: Front Plant Sci. 2018 Oct 2;9:1406. doi: 10.3389/fpls.2018.01406 (PMC6176777; doi:10.3389/fpls.2018.01406)
Supplement: Supplementary file 1 [file Table_1.DOCX]

Supplementary Table 1. Information of the 24 genotype lines included in this study.

| Diversity panel | Photoperiod classification provided | Type | Origin | Race | Flowering |
| --- | --- | --- | --- | --- | --- |
| PI_329311 | Sensitive | Energy | Ethiopia | Durra | Nonflowering |
| PI_297155 | Insensitive | Energy | Uganda | Kafir | Late August |
| PI_506069 | Sensitive | Energy | Togo | Guinea/bicolor | Nonflowering |
| PI_297130 | Sensitive | Energy | Uganda | Caudatum | Nonflowering |
| Grassl | Sensitive | Energy | Uganda | Caudatum | Late September |
| PI_152730 | Sensitive | Energy | Kenya | Caudatum/bicolor | Nonflowering |
| PI_655972 | Insensitive | Energy | South Africa | Kafir | Late August |
| PI_510757 | Sensitive | Energy | Cameroon | Durra | Nonflowering |
| PI_505735 | Insensitive | Energy | Zambia | Caudatum | Early September |
| PI_329632 | Sensitive | Energy | Ethiopia | Durra | Nonflowering |
| PI_35038 | Insensitive | Energy | United States | Caudatum | Late August |
| PI_585954 | Sensitive | Energy | Togo | Guinea | Nonflowering |
| NTJ2 | Sensitive | Energy | India | Durra | Early September |
| M81e | Sensitive | Energy | United States | Caudatum/durra | Mid September |
| PI_229841 | Insensitive | Energy | South Africa | Kafir | Mid September |
| BTx623 | Insensitive | Grain | United States | Kafir | Late August |
| Tx642 | Insensitive | Grain | United States | Kafir | Late August |
| China 17 | Insensitive | Sweet | NA | Bicolor | Mid August |
| San Chi San | Insensitive | Sweet | China | Bicolor | Mid August |
| ICSV700 | Sensitive | Sweet | NA | Bicolor | Mid September |
| Atlas | Insensitive | Sweet | United States | Durra | Late August |
| Leoti | Insensitive | Sweet | United States | Durra | Late August |
| Chinese Amber | Insensitive | Sweet | China | Caudatum/durra | Late August |
| Rio | Insensitive | Sweet | United States | Durra | Early September |
